# Antibacterial Effect of All-in-one Self-etch Adhesives on *Enterococcus faecalis*

**DOI:** 10.5681/joddd.2014.040

**Published:** 2014-12-03

**Authors:** Mohammad Esmaeel Ebrahimi Chaharom, Amir Ahmad Ajami, Mehdi Abed Kahnamouei, Elmira Jafari Navimipour, Pardis Tehranchi, Vahid Zand, Mohammad Reza Sadeghi, Aydin Sohrabi

**Affiliations:** ^1^Dental and Periodontal Research Center, Tabriz University of Medical Sciences, Tabriz, Iran; ^2^Associate Professor, Department of Operative Dentistry, Faculty of Dentistry, Tabriz University of Medical Sciences, Tabriz, Iran; ^3^Assistant Professor, Department of Operative Dentistry, Faculty of Dentistry, Tabriz University of Medical Sciences, Tabriz, Iran; ^4^Postgraduate Student, Department of Operative Dentistry, Faculty of Dentistry, Tabriz University of Medical Sciences, Tabriz, Iran; ^5^Associate Professor, Department of Endodontics, Faculty of Dentistry, Tabriz University of Medical Sciences, Tabriz, Iran; ^6^Assistant Professor, Medical Bacteriology Department, Faculty of Medicine, Tabriz University of Medical Sciences, Tabriz, Iran; ^7^Assistant Professor, Department of Orthodontics, Faculty of Dentistry, Tabriz University of Medical Sciences, Tabriz, Iran

**Keywords:** Adhesives, antibacterial agent, Enterococcus faecalis

## Abstract

***Background and aims.*** The aim of this study was to evaluate the antibacterial activity of one-step self-etch adhesives on *Enterococcus faecalis* on days 1, 7 and 14 with the use of modified direct contact test.

***Materials and methods.*** The modified directcontact test was used to evaluate the antibacterial effect of Adper Easy One, Bond Force, Clearfil S3 Bond, Futurabond M, G-Bond, iBond and OptiBond All-in-one adhesives on *Enterococcus faecalis *after aging the samples in phosphate-buffered saline for one, seven and fourteen days. Data were analyzed using one-way ANOVA and post hoc Tukey tests. Aging effect of each adhesive was evaluated by paired-sample test. In this study, P<0.05 was considered significant.

***Results.*** All the tested adhesives exhibited antibacterial activity after one day and had significant differences with the positive control group (P<0.05). After one week, OptiBond All-in-one, iBond and Futurabond M exhibited significant differences in bacterial growth from other groups (P<0.05). There were no significant differences between the groups in two weeks (P>0.05).

***Conclusion.*** iBond exhibited the highest antibacterial effect on *E. faecalis* after one week. Futurabond and OptiBond All-in-one exhibited antibacterial effects against E. faecalis for one week.

## Introduction

Long-term success of endodontically treated teeth after restorative or prosthetic treatments depends on the quality of restoration and its clinical adaptability. Historically, fiber posts have been marketed to restore endodontically treated teeth and preserve the esthetic appearance.^1^ The adhesive systems introduced for cementation of fiber posts are similar to those introduced for indirect restorations, which include the etch-and-rinse and self-etch adhesive systems. In the one-step self-etch systems, which are referred to as all-in-one adhesives, the etchant, primer and adhesive have been incorporated in one solution. Since the smear layer is not removed when these adhesives are used and functions as a component of the hybrid layer, the bacteria remaining in the smear layer might survive at tooth-restoration interface and contribute to leakage, recurrent caries, etc.^2^ Studies have shown that if tooth structure conditioners, such as primers and adhesives, have antimicrobial activity, the bacteria remaining at tooth-restoration interface might be eliminated and micro-leakage and recurrent caries can be prevented. Therefore, the antimicrobial activity of adhesives might have an important role in the longevity of restorations.^3^

The majority of studies carried out to date on the antibacterial effect of adhesive systems have evaluated their effect on *Streptococci*.^2-5^ However, *Enterococcus faecalis* is a resistant microorganism and although it is found only in a small fraction of untreated root canals, it has a major role in periradicular lesions after root canal treatment.^6^To date, only very limited number of studies have been carried out to evaluate the effect of dental adhesives on this bacterial species and the majority of studies on *E. faecalis* have evaluated the effect of materials used in root canal therapy on this bacterial species.^7-11^

 Agar diffusion test (ADT) is a common technique for the evaluation of the antibacterial effect of adhesives on various bacterial species. However, this technique has several drawbacks, including the fact that it is not an appropriate technique for water-insoluble materials and has relatively low sensitivity.^12^To overcome such drawbacks, Weiss et al^12^ introduced direct contact test (DCT) which is now commonly used in a large number of studies.^2,3^

 Considering the use of self-etch adhesives within the root canals to bond fiber posts and the importance of *E. faecalis* within the root canals and the advantages of direct contact technique, the aim of the present study was to evaluate the antibacterial effect of one-step self-etch adhesives on *E. faecalis* with the use of modified direct contact test. 

## Materials and Methods

 In the present study, seven different bonding agents in seven bonding groups, one positive control group and two negative control groups were evaluated at 24-hour, 1-week and 2-week intervals. The seven bonding groups consisted of Adper Easy one, Bond Force, Clearfil S3bond, Futurabond M, G-Bond, iBond and OptiBond All-in-one (Table 1). 

**Table 1:** One-step self-etch adhesives’ composition according to the manufacturers

Bonding agent	Manufacture	Composition
Adper Easy One	3M ESPE	Bis GMA, HEMA, Methacrylated phosphonic esters,1,6 hexanedid dimetacrylate, silica filler, Ethanol, Water, CQ
Bond Force	Tokuyama	Methacryloyloxyalkyl acid phosphate, HEMA, Bis-GMA,TEGDMA water, isopropylalco- hol, glass filler, CQ
Clearfil S Bond	Kurary	10-MDP,HEMABis-GMA, water ethanol, silanated colloidal silica, CQ
Futurabond M	VOCO	Organic acid, UDMA, HEMA, CQ,BHT
G Bond	GC	4-MET,phosphate ester monomer, UDMA, acetone, water, micro filler photo initiator
iBond	Herauskulzer	4- META, UDMA, glutaraldehyde, acetone, water, CQ
OptiBond All- in-one	Kerr	Glycerol phosphate dimethacrylate mono and difuntional methacrylate monomers,water, acetone, ethanol, CQ, filler sodium hexafluorosilicates and ytterbium fluoride

 In the present study, the modified direct contact test was used.^12^ Since it is necessary for the bacteria floating in the liquid culture media to come into contact with the bonding agent, the microplates of microbiologic tests (96-well microtiter plates) were used. 

 In each test group, based on the results of a pilot study, 32 wells were used in 7 microplates (n=32). In addition, 32 wells with the adhesives under study but without any bacteria were used as the negative control group and 32 plates with the bacterial solution but without adhesives were used as the positive control group. Another negative control group was used, which consisted of 32 wells without adhesives and bacteria, with only the culture media in order to control the sterility of microplates. 

 To begin, 25 μL of the bonding agent was placed on the wall of each well and after it was well adapted with the walls of the well, polymerization was carried out according to manufacturer’s instructions. This way, a certain volume of the space of each well was occupied by the adhesive (25 μL). Then the microplates underwent an aging process by storage in buffered phosphate saline at 37°C at 95% atmospheric moisture for 24 hours, 7 days and 14 days. During the aging process, the physiologic serum was refreshed every 24 hours. At the end of the aging process, the physiologic serum contents of the microplates were retrieved and 10 μL of *E. faecalis *(ATCC 29212) bacterial suspension (approximately 10^ 6^ bacteria) were added to each wall. The microplates were kept at 37°C for 60 minutes in a moist environment. 

 During this period, the bacteria came to direct contact with the free surface of the adhesive. Then 240 μL of BHI culture medium were added to each microplates and mixed for 2 minutes. In the final stage, serial dilutions were prepared from the content of each microtube in the BHI culture medium and 20 mL of each dilution were cultured on each solid BHI culture medium using the spread plate technique. The bacterial counts were reported as CFU/mL.^13^

 One-way ANOVA was used to evaluate results (CFU/mL means) in the seven adhesive groups and control groups at 24-hour, 7-day and 14-day intervals. HSD Tukey tests were used for the two-by-two comparisons of the groups. Aging effect of each adhesive was evaluated by paired-sample test. 

## Results

 ANOVA showed significant differences in the bacterial growth rates between the adhesives after one day and one week (P<0.001). All the tested adhesives exhibited antibacterial activity after one day and exhibited significant differences from the control positive group (P<0.05). After one week OptiBond All-in-one, iBond and Futurabond M exhibited significant differences in bacterial growth from other groups (P<0.001). iBond had more antibacterial activity than Futurabond (P=0.000) and OptiBond All-in-one (P=0.006). There were no significant differences between groups after two weeks (P>0.05). Tables 2and3 represent the number of CFU/mL in each group and the results of data analysis with SPSS 19. 

**Table 2:** Mean values of bacterial growth for different one-step self-etch adhesive systems

Bonding agent	Manufacture	Composition
Adper Easy One	3M ESPE	Bis GMA, HEMA, Methacrylated phosphonic esters,1,6 hexanedid dimetacrylate, silica filler, Ethanol, Water, CQ
Bond Force	Tokuyama	Methacryloyloxyalkyl acid phosphate, HEMA, Bis-GMA,TEGDMA water, isopropyl alcohol, glas filler, CQ
Clearfil S Bond	Kurary	10-MDP,HEMABis-GMA, water ethanol, silanated colloidal silica, CQ
Futurabond M	VOCO	Organic acid, UDMA, HEMA, CQ, BHT
G Bond	GC	4-MET,phosphate ester monomer, UDMA, acetone, water, micro filler photo initiator
iBond	Herauskulzer	4- META, UDMA, glutaraldehyde, acetone, water, CQ
OptiBond All- in-one	Kerr	Glycerol phosphate dimethacrylate mono and difuntional methacrylate monomers, water, acetone, ethanol, CQ, filler sodium hexafluorosilicates and ytterbium fluoride

**Table 3:** Pair comparisons of each bonding at different time intervals

	One day & one week	One day & two week	One week & two weeks
I Bond	-	√	√
OptiBond All-in-one	√	√	√
Clearfil S^3^Bond	√	√	-
Futurabond M	√	√	√
G-Bond	√	√	-
Adper Easy One	√	√	-
Bond Force	√	√	-
Control group	-	-	-
v are significantly different by paired samples test			

## Discussion

 Several studies have shown the effect of coronal seal of the root canal on prevention of the failure of endodontic treatment.^14-16^ The majority of these failures are due to periapical lesions as a result of microleakage and penetration of microorganisms. The odds of such lesions and failures increase when intracanal posts are used after root canal treatment because the length of canal obturation decreases after placement of a post, resulting in a decrease in the efficacy of the physical barrier between the oral cavity and periapical tissues.^17^

 Resin cements have been widely used for the cementation of intracanal posts, especially fiber posts, since such cements were introduced. Different cements have been introduced for cementation of intracanal posts, some of which are bonded using the etch-and-rinse technique and some others by using the self-etch technique. Newer versions of resin cements are self-adhesive and do not need any preparation.^18^

 Use of self-etch adhesive systems for bonding procedures has led to the elimination of the etching step and since there are no etching and rinsing steps, bacteria might remain at the tooth-restoration interface. If the primer or the adhesive in the bonding system exhibits antibacterial activity, the bacteria remaining at the interface and also the bacteria penetrating due to microleakage might be eliminated, increasing the longevity of existing restorations.^19,20^

 In the present study, the antibacterial effects of 7 types of one-step self-etch adhesive systems on *E. faecalis* were evaluated. To this end, modified direct contact test was used. Direct contact test (DCT) is a quantitative test, with an ability to test and evaluate water-insoluble materials. It is possible for microorganisms and test materials in this test to contact directly without the influence of diffusion properties of the materials under study. DCT does not rely on the size of bacteria contacting the test materials; therefore, it makes it possible to measure and evaluate standards by repeating a large number of samples, control the same plates again later, monitor and assess microbial growth and evaluate the presence and absence of the material(s) test.^4,21^

 In several studies carried out by using DCT, the culture media have been added to the walls of microplates containing the test materials and bacteria in order to evaluate bacteriostatic effects; this technique did not prevent the sustained interventional effect of the antibacterial agent on the bacteria in the culture medium. In this study, counting of CFUs was carried out immediately after the duration of time necessary for contact in order to minimize the effect of confounding factors and compare the bactericidal effect of adhesives much better and more effectively (modified direct contact test).^13^

 The antibacterial effects of 7 one-step self-etch adhesive systems were evaluated on *E. faecalis*. All the adhesives included in the present study exhibited antimicrobial effects for 24 hours and demonstrated significant differences from the positive control group, consistent with the results reported by Thai Cuchute et al,^22^ who evaluated one-bottle adhesives with the use of DCT and showed that one-bottle adhesives exhibit antimicrobial effects at least for 24 hours. The antibacterial effects of the adhesives under study might be attributed to the low pH resulting from the remaining acidic monomers.^20-23^ However, polymerization of adhesive agents results in trapping of the polymer matrix, decreasing the release of polymerizeable antibacterial components such as the acid monomers and promoting the adhesion process. Therefore, the antimicrobial and bactericidal activity of the adhesive after light-curing and polymerization of the adhesive can be explained by the fact that complete conversion of the monomer to a polymer does not take place and the remaining monomers can exert this effect.^22^ On the other hand, the monomers remaining in the oxygen inhibition layer, too, might be one of the reasons for the antibacterial activity of the adhesive.^24^

 In this study the persistence of the antibacterial effects of the adhesives was evaluated at 7-day and 14-day intervals so that their long-term effects could be evaluated. In the present study, some of the adhesives lost their antibacterial activities after 7 days; however, OptiBond All-in-one, iBond and Futurabond M adhesives preserved their antimicrobial activity and exhibited statistically significant differences from the control group and 4 other bonding groups. 

 Persistence of antibacterial activity of OptiBond All-in-one might be attributed to the fluoride content of this adhesive. Contrary to the acidic monomers which co-polymerize with the resin matrix during curing, antimicrobial agents such as fluoride can be separated from the resin matrix and diffuse out from the adhesive resin.^25^ Therefore, the fluoride in the bonding agent can function as an antibacterial agent for 24 hours and even for 1 week for this adhesive. However, there were significant differences in the antibacterial effects of this adhesive at 24-hour, 1-week and 2-week intervals and such an activity ceased after two weeks. 

 iBond preserved its antimicrobial effects for one week and no significant differences were observed between 24-hour and 1-week intervals; however, there were significant differences from the control group. Such an antibacterial effect might be attributed to the glutaraldehyde content of the adhesive, the antibacterial effect of which has been shown in previous studies.^25^ This favorable effect in the chemical structure of iBond adhesive was shown in the present study and in a study by Walter et al,^26^ who reported that iBond has antibacterial effects on various bacterial species and can maintain this effect for at least 1 week. 

 Futurabond M maintained its antimicrobial effect to some extent. BHT (butylhydroxy toluene) remaining in the structure of this adhesive might be responsible for this antimicrobial effect. Turcotte et al^27^ showed that BHT has antibacterial effects, especially against gram-positive bacteria. Since E. faecalis is a gram-positive coccus, it is possible that small amounts of BHT have eliminated it. The results of the present study showed that none of the adhesives maintained their antibacterial effects for 2 weeks. All the adhesives evaluated in the present study are methacrylate-based. Salz et al^28^ showed that in one-step self-etch adhesives with a base of methacrylate the inherent acidic environment results in the gradual destruction of these adhesives over time due to hydrolysis; however, adhesives with methacrylamide base are stable in the aqueous acidic environment. 

 In the present study, the samples were stored in phosphate-buffered saline solution to simulate aging; the solution was refreshed every 24 hours. Given the methacrylate base of all the adhesives tested, it is possible that the acidic monomers remaining in these adhesives were gradually dissolved due to continuous contact with phosphate-buffered saline solution and were destroyed. Possibly this solubility and hydrolysis resulted in a decrease in the concentration of other components of adhesives, such as iBond, Futurabond M and OptiBond All-in-one so that they could not exert their effect on bacteria; therefore, none of the bonding agents preserved their antibacterial effects for two weeks. 

## Conclusion

 The incorporation of antibacterial agents in bonding systems may affect the residual bacteria present in the root canal. Of all the bonding agents evaluated in the present study, iBond exhibited the highest antibacterial effect on *E. faecalis* after one week. Futurabond and OptiBond All-in-one exhibited antibacterial effects against *E. faecalis* for one week. None of the adhesives exhibited antibacterial activity after two weeks. 

### Aknowledgement

 The authors extend their appreciation to the office of the Vice Chancellor for Research, Tabriz University of Medical Sciences, for the financial support of this research. 
